# Leisure-time physical activity and DNA methylation age—a twin study

**DOI:** 10.1186/s13148-019-0613-5

**Published:** 2019-01-19

**Authors:** Elina Sillanpää, Miina Ollikainen, Jaakko Kaprio, Xiaoling Wang, Tuija Leskinen, Urho M. Kujala, Timo Törmäkangas

**Affiliations:** 1 Gerontology Research Center, Faculty of Sport and Health Sciences, University of Jyväskylä, P.O. Box 35 (VIV), FI-40014 Jyväskylä, Finland; 2 Institute for Molecular Medicine Finland (FIMM), University of Helsinki, Helsinki, Finland; 3 Department of Public Health, University of Helsinki, Helsinki, Finland; 4 George Prevention Institute, Department of Pediatrics, Medical College of Georgia, Augusta University, Augusta, GA USA; 5 Department of Public Health, University of Turku and Turku University Hospital, Turku, Finland; 6 Faculty of Sport and Health Sciences, University of Jyväskylä, Jyväskylä, Finland

**Keywords:** Epigenetic clock, Methylation, Twin design, Physical activity, Quantitative genetics

## Abstract

**Background:**

Epigenetic clocks may increase our understanding on human aging and how genetic and environmental factors regulate an individual aging process. One of the most promising clocks is Horvath’s DNA methylation (DNAm) age. Age acceleration, i.e., discrepancy between DNAm age and chronological age, tells us whether the person is biologically young or old compared to his/her chronological age. Several environmental and lifestyle factors have been shown to affect life span. We investigated genetic and environmental predictors of DNAm age in young and older monozygotic (MZ) and dizygotic (DZ) twins with a focus on leisure time physical activity.

**Results:**

Quantitative genetic modeling revealed that the relative contribution of non-shared environmental factors was larger among older compared with younger twin pairs [47% (95% CI 35, 63) vs. 26% (95% CI: 19, 35), *p* < 0.001]. Correspondingly, genetic variation accounted for less of the variance in older [53% (95% CI 37, 65)] compared with younger pairs [74% (95% CI 65, 82)].

We tested the hypothesis that leisure time physical activity is one of the non-shared environmental factors that affect epigenetic aging. A co-twin control analysis with older same-sex twin pairs (seven MZ and nine DZ pairs, mean age 60.4 years) who had persistent discordance in physical activity for 32 years according to reported/interviewed physical-activity data showed no differences among active and inactive co-twins, DNAm age being 60.7 vs. 61.8 years, respectively [between-group mean-difference: − 1.17 (95%CI − 3.43,1.10)]. Results from the younger cohort of twins supported findings that LTPA is not associated with DNAm age acceleration.

**Conclusions:**

In older subjects, a larger amount of variance in DNAm age acceleration was explained by non-shared environmental factors compared to young individuals. However, leisure time physical activity during adult years has at most a minor effect on DNAm age acceleration. This is consistent with recent findings that long-term leisure time physical activity in adulthood has little effect on mortality after controlling for genetic factors.

## Introduction

Advances in the fields of molecular biology have produced novel promising candidate biomarkers and their combinations that may be considered as biological aging clocks. These clocks may increase understanding on human aging and how genetic and environmental factors regulate the biological aging process during a life span [1]. So far, one of the most promising new aging clocks is DNA methylation (DNAm) age, developed by Steve Horvath, and also known as “epigenetic clock” [2].

DNAm is one type of epigenetic modification that affects reading and packing of genetic information. DNAm age is a multi-tissue age estimate based on DNA methylation at 353 specific age-related CpG sites. It is determined with a special algorithm, which is publicly available (https://dnamage.genetics.ucla.edu/). DNAm age increases over chronological age, but it is not yet clear if it is only a marker of biological aging or has an effect on aging per se [2]. The epigenetic clock appears to be associated with a wide spectrum of aging outcomes, most consistently mortality [1, 3]. Discrepancy between DNAm age and chronological age, i.e., higher “age acceleration” predicts all-cause mortality above and beyond chronological age and traditional risk factors such as body mass index (BMI), education, physical activity, alcohol use, smoking, and certain comorbidities [4]. High age acceleration is also associated with multiple aging phenotypes including poorer cognitive performance, lower grip strength, poorer lung function [5], and increased frailty index [6], BMI, and indicators of the metabolic syndrome [7]. The unresolved questions related to the epigenetic clock are if and how the clock’s ticking rate is modifiable and whether the methylation changes seen with age and aging phenotypes actually drive the phenotypes or whether they merely represent the workings of intrinsic control mechanisms [8].

It is clear that variation observed in aging and especially longevity has a genetic component [9]. Comparison within monozygotic (MZ) twin pairs has suggested that the ticking rate of the epigenetic clock is also largely modified by genetic factors [2]. However, simple correlations among MZ twins can only account for the upper limit of genetic component, and more sophisticated statistical techniques are needed to partition total variance of DNAm age into genetic and shared and non-shared environmental components of variance.

So far, it is also not clear whether the genetic component in variation of DNAm age changes over a life span. On the other hand, some environmental exposures and behaviors such as infections, diet, alcohol use, smoking, and work exposures predispose to age-related diseases and increase probability of death. Only part of individual variation to life expectancy can be accounted for using known and measured characteristics and exposure. An epigenetic clock could provide insights into the mechanisms behind why some individuals age faster than others and are more prone to age-related diseases and accelerated decline in physical function.

Physical activity is a potentially modifiable behavior that could slow down the rate of cellular and molecular damage accumulation and blunt the decline in physiological function with increasing age. Various mechanisms which mediate the association between high leisure-time physical activity and reduced risk of various non-communicable diseases have been suggested [10, 11]. High physical activity in older age is associated with better health, longer life span, and better quality of life [12]. However, it is unclear whether physical activity affects life span after controlling for genetic factors [13]. Twin models are able to provide evidence for the causality of environmental influences, including physical activity. As MZ twins share, in practice, the same genome, any phenotypic differences within MZ pairs are solely due to non-shared environmental differences and are not confounded by genetic variation.

The purpose of the study was to estimate the magnitude of genetic and environmental factors affecting variation in DNAm-based age acceleration in young and older participants recruited from Finnish twin cohort using quantitative genetic modeling. In addition, we used powerful co-twin control analysis to test the hypothesis that long-term leisure-time physical activity is one of the environmental factors that affect variation in DNAm age in older age.

## Results

### Characteristics of the twin cohort

Young MZ twin pairs were slightly younger compared to (dizygotic) DZ twin pairs, while there were no differences in chronological or DNAm age in older MZ and DZ pairs (Table 1). Mean age acceleration was similar among young and older twin pairs, whether MZ or DZ. Young DZ twins were more likely to be current smokers when compared to young MZ twins.

**Table 1:** Characteristics of the young and older monozygotic and dizygotic individual twins participating into the current study

	Young 20–25 years		MZ vs DZ difference	Older 55–70 years		MZ vs DZ difference
	MZ	DZ	*p* value	MZ	DZ	*p* value
Chronological age (years)	22.7 (0.9)	22.4 (0.7)	0.005	61.4 (3.7)	62.1 (3.8)	0.29
DNAm age (predicted years)	22.2 (3.8)	21.3 (3.6)	0.015	61.2 (5.7)	62.6 (5.4)	0.096
Age acceleration (residuals)	0.29 (3.54)	− 0.40 (3.48)	0.065	− 0.02 (5.46)	0.65 (4.95)	0.35
Body mass index (kg/m^2^)	23.0 (3.5)	23.7 (4.3)	0.055	27.2 (5.0)	28.2 (4.7)	0.11
Women, *n* (%)	218 (65%)	142 (59%)	0.28	162 (66%)	44 (52%)	0.11
Never smokers, *n* (%)	177 (53%)	126 (52%)	0.001	117 (48%)	42 (50%)	0.95
Former smokers, *n* (%)	30 (9%)	17 (7%)	–	86 (35%)	29 (35%)	–
Current smokers, *n* (%)	101 (30%)	98 (40%)	–	41 (17%)	13 (15%)	–

Values are means and standard deviations unless otherwise stated. MZ, monozygotic; DZ dizygotic. Differences between groups on continuous variables were tested on design corrected *T*-test for independent samples and chi-square test (sex, smoking)

Correlation between chronological age and DNAm age was 0.65 (*p* < 0.001) in participants under 40 years and 0.53 (*p* < 0.001) in participants over 50 years old.

### Genetic modeling

In age acceleration, higher ICCs, i.e., greater within pair similarity, were observed in young [0.74 (0.66, 0.80)] and older [0.59 (0.46, 0.69)] MZ twin pairs compared to young same-sex [0.43 (0.27, 0.56)], opposite-sex DZ [0.35 (0.16, 0.52)], and older same-sex [0.17 (− 0.13, 0.45)] DZ twin pairs.

Analysis revealed that a model including additive genetic effects and unique environment (AE) showed best fit for age acceleration data both in young and older subjects. Genetic factors explained more of the variation in DNAm-based age acceleration in young [74 (65, 82)%] compared with older twin pairs [53 (37, 65)%] (Table 2). Correspondingly, the unique environmental component had a larger estimate in older twin pairs [47 (35, 63%)] compared to young twin pairs [26 (19, 35)]%, with a significant difference between age groups (*p* < 0.001).

**Table 2:** Best fitting models for DNAm age acceleration in young and older twins

	Additive genetic (*a*^2^)	Non-shared environment (*e*^2^)	Sex (men vs. women)	BMI	Smoking (former vs. never)	Smoking (current vs. never)
DNAm age acceleration						
Unadjusted (AE)						
Young	0.74* (0.65, 0.81)	0.26* (0.19, 0.35)	–	–	–	–
Older	0.58* (0.45, 0.69)	0.42* (0.31, 0.55)	–	–	–	–
Adjusted (AE)						
Young	0.74* (0.65, 0.82)	0.26* (0.19, 0.35)	0.61 (− 0.16, 1.37)	0.065 (− 0.01, 0.13)	− 0.23 (− 1.09, 0.64)	− 0.17 (− 0.79, 0.47)
Older	0.53* (0.37, 0.65)	0.47* (0.35, 0.63)	3.29* (2.00, 4.54)	0.07 (− 0.03, 0.17)	0.68 (− 0.32, 1.73)	0.53 (− 0.84, 1.96)

Data are proportion of total variance (95% CI) adjusted for sex, smoking and BMI. *p < 0.001 for young vs. old

### Discordant twin analysis

Main descriptive characteristics in TWINACTIVE cohort are shown in Table 3. There was a long-term high-volume pairwise difference in leisure-time physical activity between the active and inactive co-twins. Although there were differences in the body composition between active and inactive co-twins, BMI did not differ statistically significantly.

**Table 3:** Descriptive characteristics of the TWINACTIVE cohort (MZ *n* = 7 and DZ = 9 twin pairs) at the time DNA sample for methylation analysis was taken (18)

	Inactive	Active	*p* value
Sex (female/male)	5/11		
Age (years)	60 (50–74)	–	–
Body height (cm)	171.8 (10.4)	171.1 (9.9)	0.39
Body weight (kg)	79.5 (18.4)	72.9 (11.9)	0.12
Body mass index (kg/m^2^)	26.7 (3.5)	24.8 (2.6)	0.09
Leisure-time MET index (MET hour/day)	1.6 (1.4)	8.4 (4.1)	< 0.001

Differences between groups were tested by Wilcoxon matched-pair signed-rank test

**Fig. 1:**
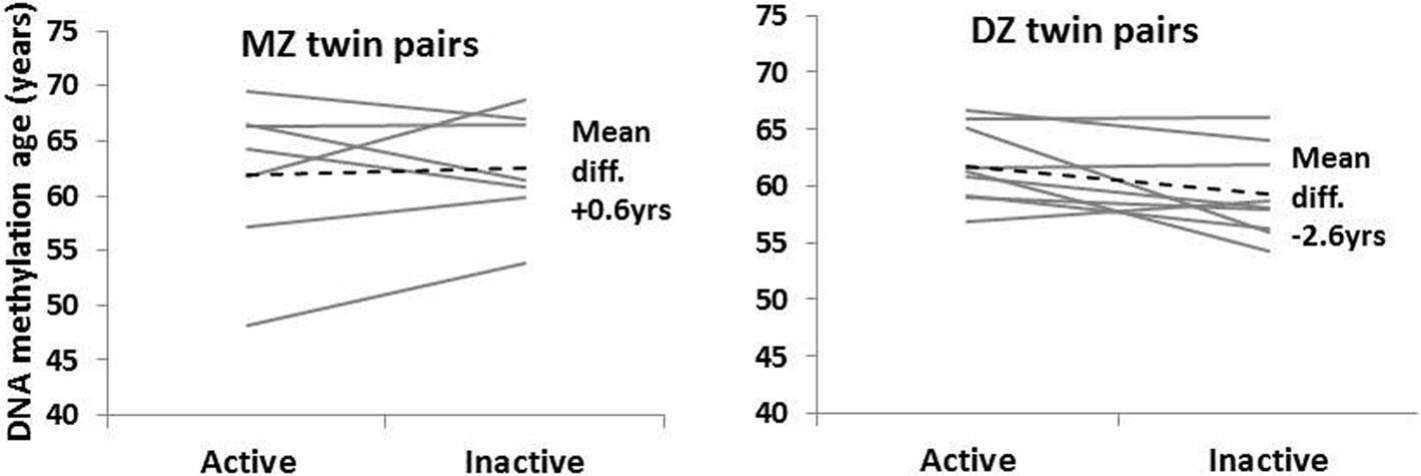
DNA methylation age in active and inactive monozygotic (MZ) and dizygotic (DZ) twin pairs. Dashed line represents the mean within-pair difference in DNA methylation age. Other lines represent individual pairs.

Among the leisure-time physical activity discordant twin pairs, DNAm age did not differ between the active and inactive co-twins, being 60.7 vs. 61.8 years, respectively [between-group mean difference: − 1.17 (95%CI − 3.43,1.10)]. There were also no differences within the pairs, when MZ and DZ twin pairs were analyzed separately [mean differences for MZ 0.62 (95%CI − 3.7, 4.9) and DZ − 2.6 (95%CI − 5.3, 0.2)] (Fig. 1)), with no systematic differences for individual pairs.

#### Replication

Associations between LTPA and DNAm age acceleration were also investigated in younger cohort of Finnish twins (*n* = 683, mean age 22.4 years). The level of physical activity at the same time point with DNA extraction was assessed with the Baecke questionnaire, yielding three indexes: sport index, leisure-time index, and work index [14]. Work index refers to physical activity at work, sport index to sports participation during leisure time, and leisure-time index to physical activity during leisure time excluding sport activities [14]. We found no associations between physical activity indexes and DNAm age acceleration in this age group (*R*^2^ < 0.005, *p* = n.s. in all associations, i.e., DNAm age acceleration and work index, leisure index, and sport index). In addition, we identified physical activity discordant twin pairs with respect to sport index. We were able to identify only 31 pairs with within-pair difference > 1 h per day, i.e., one of the twins having a significantly lower activity level compared to the other twin of the pair. Of these pairs, eight were monozygotic (MZ), 11 opposite-sex dizygotic (DZ), and 12 same sex DZ pairs. Both twins of a pair showed highly similar age acceleration (in all 31 pairs: *r* = 0.039 SE 0.48, *p* = 0.936). Within-pair and sex-adjusted *p* values for differences between active and inactive twins were *p* = 0.641 (MZ), *p* = 0.999 (same sex DZ), and *p* = 0.676 (opposite sex DZ).

## Discussion

Since Horvath’s epigenetic clock was published, we have seen that it is likely genetically regulated as the ticking rate of the epigenetic clock within co-twins of MZ twin pairs seems to be highly correlated [2]. However, the within-pair correlations in MZ twin pairs provide only an upper limit to the heritability, where the relative roles of genetic and shared environmental factors, epigenetic alterations, and complex gene-gene or gene-environment-interactions cannot be teased apart. Within-pair similarity of MZ twins is not only due to shared genetic factors, but may also reflect common fetal or early childhood environmental factors, as the co-twins often share the same early environment. It may also reflect later experiences and exposures, such as lifestyle but also hobbies, and occupational and residential exposures that MZ twins share more often than DZ pairs on average [15]. Both MZ and DZ twin pairs are needed to calculate the relative contributions of genetic, shared environmental, and non-shared environmental factors on variation in DNAm age acceleration. We showed by applying quantitative genetic modeling methods, that genetic factors explain a majority of variance in DNAm age acceleration in young individuals, but that environmental exposures have also a significant age-dependent role in the epigenetic aging process. Based on our results, both genetic and environmental factors seem to have almost equal effect on age acceleration in older age. No effect of the early environment was seen in the adult pairs, suggesting that such effects, if present, are not sustained into adulthood. On the other hand, the power of the twin design to detect common environmental effects is less than the power to detect genetic effects [16].

Epidemiological studies are prone to selection bias caused by genes or other childhood familiar factors while investigating associations between environmental exposure and progress of aging process, or morbidity/mortality. Co-twin-control study is a unique study design, which can be used to investigate the effects of long-term physical activity on epigenetic aging, with both genetic and familial factors standardized. With data from the TWINACTIVE cohort, we were able to investigate if high-volume leisure-time physical activity is one of the environmental factors that affects variation in DNAm age acceleration in older age. In the TWINACTIVE cohort, the mean intrapair difference in leisure-time physical activity (8.8 MET hours/day) during the 32-year follow-up period corresponds to a volume of a light 2-h daily walk. As MZ twin pairs share all their segregating genotypes, it can be hypothesized that any intrapair difference between the co-twins is due to the difference in environmental factors (including physical activity) and possible epigenetic modifications caused by the environmental exposures and experiences. The leisure-time physical activity discordant twin pairs differed by peak exercise capacity, knee extension strength, body composition (bone structure, fat free mass, body fat distribution), structure of the heart, metabolic pathways and profile, liver fat, gene expression in fat and muscle tissue, etc. [17]. These exercise-related positive alterations in body composition and function are known to help in prevention of several cardiovascular and other inactivity-related diseases, which are the main causes of mortality. Despite all phenotypic differences between the inactive and active co-twins, we did not see any differences in DNAm age acceleration, i.e., faster or slower biological aging.

Twin pairs with leisure-time physical activity discordance over three decades were used in this study, resulting in limited sample size, which in turn may reduce the credibility when generalizing our results to the general population. It must also be noted that although the discordance covered a very large age span, we cannot exclude the possibility that early life health habits or the amount of physical activity may have biased our results. We did not have data on the twins’ physical activity patterns in childhood, but in our experience it is difficult to identify MZ pairs discordant for physical activity during childhood and adolescence. Thus, the discordance arises generally only once the twins leave their childhood home. In utero and during infancy and childhood, the organismal growth accompanied with the high number of cell divisions leads to logarithmic ratio of epigenetic age and chronological age, i.e., faster ticking clock compared to adult age [2]. It has been suggested that the ticking rate of epigenetic clock is largely set before adulthood and remains constant thereafter [18]. However, our findings from genetic modeling show that the relative effect of environmental factors is larger in older twins and thus does not support the hypothesis above. Marioni et al. [19] showed recently in six European cohorts that DNAm age increases at a slower rate than chronological age, which may indicate stronger influence on environmental factors, but also survival bias, ceiling effects of age acceleration at older age plateau, or other factors related to the underlying training population of Horvath’s epigenetic clock. In addition, environmental factors may have different effects on epigenetic aging during adult age. Nevalainen et al. observed an association between increased BMI and accelerated epigenetic aging in the blood cells in middle-aged individuals, but not in young adults or nonagenarians [20]. It is clear that larger longitudinal studies are needed to elucidate whether the ticking rate of the epigenetic clock remains constant over an adult life span or whether certain periods of time (puberty, menopause, diseases, etc.) or exposures result in periods of a faster or slower ticking rate.

We identified a fairly high correspondence between DNAm age and chronological age in both age groups of twins that we studied. Similar strong linear relationships with chronological age and DNAm age have been reported earlier in other large cohorts [3, 21]. The relatively high precision with chronological age together with a number of studies showing associations with aging phenotypes and mortality [5, 22] has already demonstrated that DNAm age is a robust biomarker of age. However, it is not known what DNAm age truly measures [2, 22]. Further studies are required to establish whether DNAm actually regulates aging or whether it is just a biomarker that correlates highly with chronological age.

In this study, DNAm age was analyzed using blood samples rather than muscle tissue, which may be a more relevant tissue in terms of physical activity. Although DNAm age in muscle tissue and chronological age has modest to high correlations compared to blood, muscle tissue may have lower correspondence with chronological age [2, 22]. There is also some evidence that DNAm age may vary within the same individual depending on the tissue sampled [2]. Aging of the liver, rather than blood, muscle, or fat tissue, is accelerated in obese subjects [22], and each tissue may have its own aging profile. Long-term physical activity produces numerous adaptive metabolic and structural responses directly to muscle tissue. However, we are not aware of studies that have investigated DNAm age of the muscle tissue in association with physical activity. Future studies examining DNAm age in muscle tissue may enlighten if leisure-time physical activity affects aging of the muscle tissue and subsequently age-related decline in physical functioning.

In conclusion, we provided further evidence for a role of genes and environmental factors in controlling biological aging. The relative contribution of genes versus environment on epigenetic age acceleration exhibited an age dependency with a significantly greater relative impact of the environment among older compared with younger twins. Although the impact of leisure-time physical activity on health, well-being, and older age physical functioning is well documented [23–25], our genetically controlled co-twin-control study did not provide evidence that long-term high-volume physical activity in adult age slows down or accelerates biological aging; the relatively small sample size cautions against drawing far-reaching conclusions from these results. While accelerated aging detected by Horvath’s clock is clearly associated with increased mortality risk [3, 4, 26], our results are consistent with the findings that leisure-time physical activity in adult age has little or no effect on mortality after controlling for genetic factors [13]. It is possible that genetic pleiotropy affecting exercise participation might partly explain the frequently observed associations between physical activity and reduced mortality in humans [13]. Whether physical activity affects programming of the epigenetic clock ticking rate in childhood, or younger adulthood or has tissue specific differences in ace acceleration requires further studies.

## Materials and methods

### Participants and study design

The study sample was part of the Finnish Twin Cohort, which includes three large cohort studies: (1) the older twin cohort of twins born before 1958, (2) Finntwin16, born in 1975–1979, and (3) Finntwin12, born in 1983–1987 [27]. All MZ and DZ twin pairs who had taken part in several clinical in-person studies with sampling for whole blood DNA and subsequent epigenetic methylation analyses were included into baseline correlative analysis (*n* = 1249 twin individuals aged 20 to 72 years). Same-sex and age-range young (20- to 25-year-old) and older (55- to 70-year-old) MZ (*n* = 168 young, *n* = 122 older) and DZ twin pairs (*n* = 121 young, *n* = 42 older) were selected for genetic modeling (Table 1). Within pair correlation in DNAm age was also calculated for young opposite-sex DZ pairs (*n* = 93).

*Physical activity discordant twin pairs* initiated from the older cohort of a Finnish twin cohort [27–29]. The comprehensive identification process of the twin pairs has been described in detail by Leskinen [30]. Briefly, first physical activity assessments were carried out during 1975 and 1981 [31]. All types of leisure time and commuting-related physical activity reported in standardized questionnaires were taken into account in determining the discordance of twin pairs. Leisure-time activity was quantified as metabolic equivalent [intensity × duration × frequency] and expressed as a sum score of leisure time MET hour/day. Twin pairs whose difference in volume of the physical activity were > 3 MET hours/day were invited to the retrospective follow-up interviews on leisure activity (covering the years from 1980 to 2005 in 5-year intervals), which were carried out during years 2005–2007 [32]. The cut-off point for discordance was two MET hours per day. Pairs with consistent physical activity discordance over a 32-year follow-up period were invited to participate in the TWINACTIVE study [32]. Finally, after the comprehensive screening process, of the 5663 originally healthy same-sex twin pairs, 16 same-sex middle-aged and older (age range 50–74 years) twin pairs (7 monozygotic and 9 dizygotic same-sex pairs, total 5 female pairs) participated in the study measurements (Table 2).

### DNA methylation age and age acceleration

High molecular weight white blood cell DNA was extracted using QIAamp DNA Mini Kit (QIAGEN, Nordic, Sollentuna, Sweden). Bisulfite conversion of DNA was completed using EZ-96 DNA methylation-Gold Kit (Zymo Research, Irvine, CA, USA) according to the manufacturer’s instructions, and the co-twins were always converted on the same plate to minimize potential batch effects. Genome-wide DNAm was measured using Illumina’s Infinium HumanMethylation450 BeadChip and the Infinium MethylationEPIC BeadChip (LTPA discordant twins), according to the manufacturer’s instructions (Illumina, San Diego, CA, USA). The Illumina BeadChips measure single-CpG resolution DNAm levels across the human genome. CpG site (probe) intensities were transformed to beta values with a standard equation in which beta is the ratio of the methylated probe (*m*) intensities to the overall intensities (*m* + *u* + *α*, where *α* is the constant offset, 100, and *u* is the unmethylated probe intensity). The resulting beta values ranged from 0 (completely unmethylated) to 1 (fully methylated). DNAm age was calculated using a validated algorithm and online tool (https://dnamage.genetics.ucla.edu [2]) which is based on 353 specific CpG sites measured on the 450 BeadChip and known to associate with aging based on their DNA methylation. Approximately 21,000 probes common to both the Illumina 27K and 450K arrays were imputed into the online program [2]. CpGs that were not present on the EPIC BeadChip (*n* = 20) were coded missing (NA) in all LTPA discordant twin samples, and the raw beta values of CpG probes were used as the input for calculating the epigenetic age. Potential batch effects were corrected and beta values normalized using the calculator’s internal BMIQ-based method. In the normalization step, the beta value distributions of the submitted data are fitted to the distributions of the training data set used to build the calculator. Horvath’s DNAm age predictor is based on data from multiple tissue types, so it is robust to differences in white blood cell counts. Therefore, adjustment for cell proportions was not used [19]. However, we experimented adjusting for cell types [2, 33], that it did not affect our main results in heritability analysis. Age acceleration, which describes the difference between chronological age and DNAm age (“faster or slower biological aging”), was calculated for all subjects as the residuals from a linear regression model of DNAm age on chronological age.

*Adjusting factors* included sex, smoking (never, former, current), and body mass index (BMI, kg/m^2^), which was calculated from measured or self-reported weight and height.

### Statistics

Baseline statistics was performed by Stata 14 software (StataCorp, Inc., College Station, TX, USA). Data are shown as means and standard deviations unless otherwise stated. Associations between chronological age and DNAm age were analyzed using standardized regression coefficients to represent bivariate correlations. *p* values for differences between individuals from the MZ versus DZ twin pairs were derived from design-corrected Student’s *t* test for independent samples or chi-square test (Williams, Biometrics 2000). Intraclass correlation coefficients (with their 95% CIs) were computed separately for the MZ and DZ twin pairs to estimate the level of within-pair similarity and the ratios of the MZ and DZ correlations.

Quantitative genetic modeling is based on assumption that greater similarity observed between MZ twin pairs compared to DZ twin pairs is evidence for genetic influence on the trait because MZ twins share fully their genomic sequence while DZ twins share on average 50% of their segregating genotypes [34]. Genetic modeling was conducted in a four-group model separating the two zygosity and age groups using standard univariate twin modeling based on linear structural equations [34]. The applied model is based on the assumption that phenotypic variation can be decomposed into additive genetic (A) and genetic dominance (D) or shared environmental (C) and nonshared (unique) environmental effects (E) [35]. Additive genetic (A) effects result from single gene effects added over multiple loci, whereas dominant genetic factors (D) refer to genetic interaction within the same locus. Common environment (C) refers to environmental factors shared by twins reared in the same family, and unique environment (E) represents the environmental experiences and exposures that are unique for the individual twin. To determine the best fitting model, full sequences of models from the ACE- and ADE-component arms were fitted to the data. The significance of the contribution of individual variance components to the total trait variance was tested against a full model using submodels in which one or more of the variance components were fixed to zero according to the standard procedure [36]. The likelihood ratio test and information criteria were used to identify the most parsimonious and best-fitting model to explain the observed pattern of twin similarity in the young and older MZ and DZ twin pairs and to compare the difference in model fit between the models. Genetic dominance effects in the absence of additive effects (DE model) were considered unsupported by the twin design and were not fitted. For older twin pairs, we tested also sibling interactions model, which had nonsignificant impact on the results (*p* = 0.843). All models were also adjusted for main effects of sex, smoking, and BMI, which are known to affect DNA methylation and biological aging. Differences in fit between models in young and older twin pairs as well as the component structure were tested using the likelihood ratio test for nested models and information criteria for non-nested models. Analyses were performed with MPlus 7.

Comparison of DNAm age within leisure-time physical activity same-sex discordant twin pairs was done by the Wald test (*t* test adapted for clustered twin data) for independent samples, while adjusting for chronological age and sex.

## Abbreviations

BMI: Body mass index
CpG: Cytosine nucleotide followed by a guanine nucleotide
DNAm: DNA methylation
DZ: Dizygotic
ICC: Intraclass correlation coefficient
LTPA: Leisure time physical activity
MET: Metabolic equivalent
MZ: Monozygotic
